# Independent Mechanisms Target SMCHD1 to Trimethylated Histone H3 Lysine 9-Modified Chromatin and the Inactive X Chromosome

**DOI:** 10.1128/MCB.00432-15

**Published:** 2015-10-30

**Authors:** Nicholas J. Brideau, Heather Coker, Anne-Valerie Gendrel, C. Alistair Siebert, Karel Bezstarosti, Jeroen Demmers, Raymond A. Poot, Tatyana B. Nesterova, Neil Brockdorff

**Affiliations:** a Department of Biochemistry, University of Oxford, Oxford, United Kingdom; b Oxford Particle Imaging Centre, Wellcome Trust Centre for Human Genetics, University of Oxford, Oxford, United Kingdom; c Proteomics Center, Erasmus MC, Rotterdam, The Netherlands; d Department of Cell Biology, Erasmus MC, Rotterdam, The Netherlands

## Abstract

The chromosomal protein SMCHD1 plays an important role in epigenetic silencing at diverse loci, including the inactive X chromosome, imprinted genes, and the facioscapulohumeral muscular dystrophy locus. Although homology with canonical SMC family proteins suggests a role in chromosome organization, the mechanisms underlying SMCHD1 function and target site selection remain poorly understood. Here we show that SMCHD1 forms an active GHKL-ATPase homodimer, contrasting with canonical SMC complexes, which exist as tripartite ring structures. Electron microscopy analysis demonstrates that SMCHD1 homodimers structurally resemble prokaryotic condensins. We further show that the principal mechanism for chromatin loading of SMCHD1 involves an LRIF1-mediated interaction with HP1γ at trimethylated histone H3 lysine 9 (H3K9me3)-modified chromatin sites on the chromosome arms. A parallel pathway accounts for chromatin loading at a minority of sites, notably the inactive X chromosome. Together, our results provide key insights into SMCHD1 function and target site selection.

## INTRODUCTION

SMCHD1 is a noncanonical member of the SMC family of chromosomal proteins that plays an important role in X chromosome inactivation in mammals (1–3). *Smchd1* loss of function results in early lethality in female embryos, attributable to the derepression of ∼10% of genes on the inactive X chromosome (Xi) (4, 5). This effect has been linked to hypomethylation of Xi CpG islands (CGIs) (6) and a deficiency in Xi chromatin compaction (7). In addition to its role in X inactivation, SMCHD1 is important for silencing at repeat sequences, several imprinted gene clusters, and also the monoallelically regulated protocadherin gene cluster (4, 5). Similar to Xi, the SMCHD1 function at these loci is linked to a loss of DNA methylation. Recently, mutations in human SMCHD1 have been shown to underlie type 1 and type 2 facioscapulohumeral muscular dystrophy (FSHD) (8–10), with both types of the disease being dependent on the epigenetic silencing function of SMCHD1 at the D4Z4 repeat sequence. Beyond its role in gene repression, SMCHD1 has been shown to be involved in double-strand-break repair in plants (11) and in nonhomologous end joining in mammalian cells (12, 13).

While progress has been made toward defining biological roles for SMCHD1, relatively little is known about the biochemical properties of this protein and how these properties relate to SMCHD1 localization and function at target loci. SMCHD1 is a large protein, ∼230 kDa, and the major conserved domains are a carboxy-terminal SMC hinge domain (HD), which is flanked by short coiled-coil regions, and an amino-terminal GHKL ATPase domain. There is also a region with weak homology to the bromo-adjacent homology (BAH) domain located near the GHKL ATPase domain (14). In a recent study, human SMCHD1 was identified as an interactor of the protein HBiX1, which in turn interacts with human heterochromatin protein 1 (HP1) paralogs (7).

In this study, we have applied proteomic, biochemical, and molecular analyses to better understand the mechanism of action of SMCHD1. Proteomic screening revealed that SMCHD1 interacts with LRIF1, the mouse homolog of HBiX1, and with HP1 protein paralogs. No major stoichiometric interaction partners were identified. We show that SMCHD1 homodimerizes, primarily through the SMC hinge domain, and that the GHKL domain is active in hydrolyzing ATP. Electron microscopy (EM) studies show that SMCHD1 homodimers form aligned rod-like structures with globular regions at either end, similar to canonical prokaryotic and eukaryotic SMC protein complexes. We further show that an indirect interaction mediated by the LRIF1 and HP1 proteins loads SMCHD1 onto chromatin marked by trimethylation of histone H3 lysine 9 (H3K9me3). The GHKL ATPase activity and the BAH domain are not required for the interaction with H3K9me3, but both are required for SMCHD1 localization to Xi that occurs independently of the H3K9me3/LRIF1/HP1 pathway.

## MATERIALS AND METHODS

### Cloning and mutagenesis.

*Smchd1* was PCR amplified from cDNA from a 129 background and cloned into either the pcDNA3 vector with a C-terminal hemagglutinin (HA) epitope or the pCBA-Tag1 vector with a C-terminal double-FLAG epitope. Subsequent mutagenesis was performed on both HA- and FLAG-tagged Smchd1 plasmids. The QuikChange Lightning kit (Agilent) and the primers listed in Table 1 were used to introduce the point mutations E147A and G1872A/G1875A/G1876A according to the manufacturer's protocol. Deletion of the BAH domain was performed by annealing oligonucleotides dBAH_F and dBAH_R (Table 1) and ligating the construct between the KpnI and PflMI restriction sites. Deletion of the hinge domain was accomplished by digesting *Smchd1* plasmids with BsrGI and religating the digested plasmid. *Lrif1* was cloned by reverse transcription-PCR (RT-PCR) of cDNA from wild-type (WT) E14 cells, and the sequence was verified. *Lrif1* cDNA was cloned by ligation-independent cloning (LIC) into pCAG-eGFP or pCAG-mCherry to generate N-terminal fusion proteins.

**TABLE 1:**
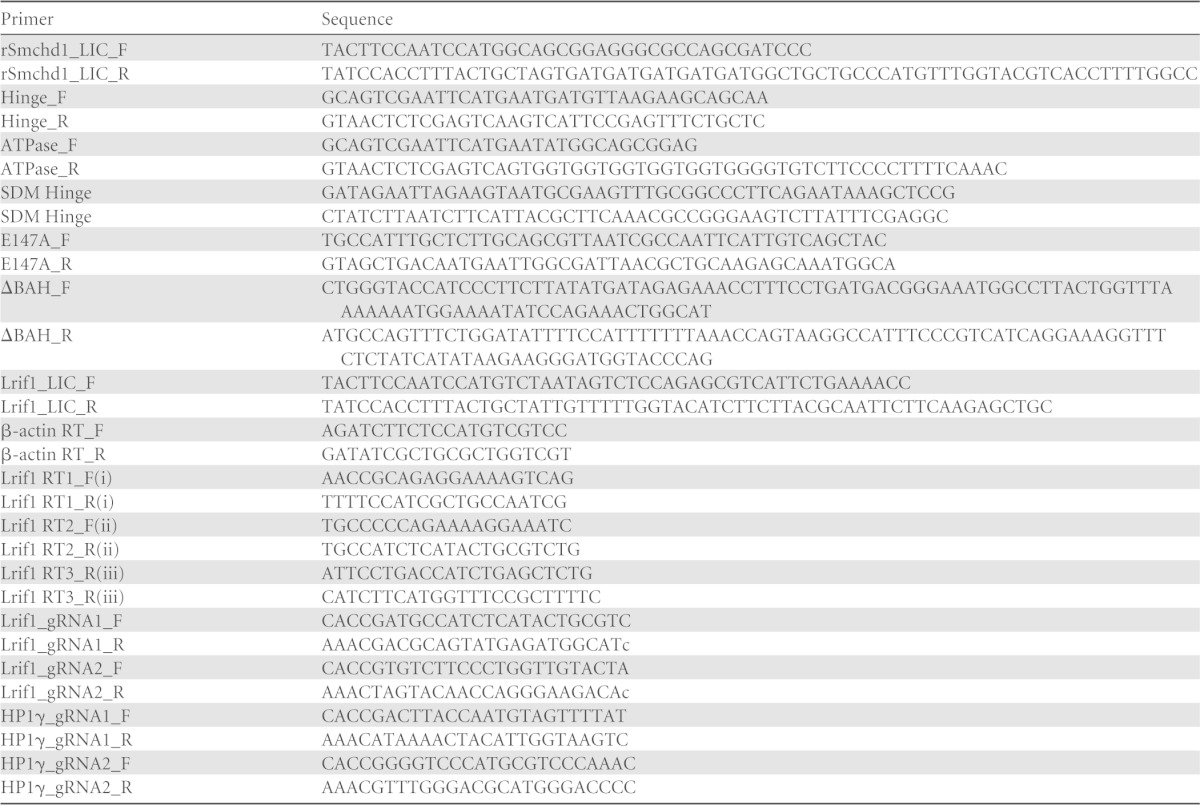
Oligonucleotide sequences

### Protein expression and affinity purification.

Full-length FLAG-tagged recombinant SMCHD1 (rSMCHD1) was expressed by using baculovirus and purified from Sf9 cells. Sf9 cells were cultured in SF900 II serum-free medium (Invitrogen) at 27°C. Sf9 cells at 1.5 ×10^6^ cells/ml were infected with Smchd1 P3 virus for 48 h. Cells were consequently harvested, washed in ice-cold phosphate-buffered saline (PBS), pelleted again, snap-frozen, and stored at −80°C. Cell pellets were resuspended in lysis buffer F (10 mM Tris [pH 8.0], 500 mM NaCl, 4 mM MgCl_2_, 2.0 mM dithiothreitol [DTT], 20% glycerol, complete protease inhibitor) and homogenized in a tight-fitting Dounce homogenizer 30 times. Lysates were spun at high speed, and the supernatant was diluted and mixed with anti-FLAG M2–agarose beads (Sigma) for 4 h at 4°C with rotation. Recovered beads were washed in wash buffer F (10 mM Tris [pH 8.0], 150 mM NaCl, 2 mM MgCl_2_, 1 mM DTT, 15% glycerol) three times, and FLAG-SMCHD1 was eluted with 0.2 mg/ml FLAG peptide in wash buffer F. The hinge domain of SMCHD1 was cloned into pET28a (Novagen) and transformed into BL21-RosettaBlue cells (Novagen). Expression was induced by the addition of 0.1 mM isopropyl-β-d-thiogalactopyranoside (IPTG) for 5 h at 30°C. Cells were harvested and lysed by sonication in lysis buffer (20 mM Tris-HCl [pH 8.0], 250 mM NaCl, 0.1% NP-40). His-tagged SMCHD1 hinge protein was purified form the soluble bacterial extract by using Talon resin (Clontech) and eluted from the resin with 250 mM imidazole. Eluents were pooled and dialyzed in a solution containing 50 mM HEPES (pH 7.9), 100 mM KCl, 10% glycerol, and 0.5 mM DTT. The ATPase domain was cloned into pMAL-c2x, transformed, expressed, and lysed as described above for the hinge domain. Cell lysates were mixed with amylose resin (NEB) and eluted from the resin with 10 mM maltose. Eluents were pooled and dialyzed as described above.

### Size exclusion chromatography (SEC).

Nuclear extracts (NEs) from embryonic stem cells (ESCs) were applied to a Superose 6 column (GE Healthcare) for separation of SMCHD1 complexes and collected in 52 fractions of 0.25 ml. Affinity-purified FLAG-rSMCHD1 was further purified by using a Superose 6 column with wash buffer F. For the hinge gel filtration experiment, affinity-purified wild-type and mutant hinges were applied to a Superdex 75 10/300 column (GE Healthcare) and collected in 0.5-ml fractions.

### ATPase assay.

An ATPase activity assay was performed by using the PiLock Gold kit (Innova Biosciences) with 1.0 μM protein in reaction buffer (125 mM NaCl, 50 mM Tris [pH 7.5], 5.0 mM MgCl_2_, 1.0 mM ATP) in a final volume of 50 μl for 60 min at 37°C. A standard curve was generated by using the phosphate standards in the kit, and activity was calculated according to the manufacturer's instructions. For the ATPase assays with radicicol, the compound was resuspended in dimethyl sulfoxide (DMSO) and diluted to the concentrations listed in Fig. 4D.

### Sucrose gradient analysis.

Five micrograms of purified FLAG-rSMCHD1 or 100 μg of nuclear extract, 30 μg of ovalbumin (43 kDa) and conalbumin (67 kDa), and 50 μg of aldolase (158 kDa), ferritin (443 kDa), and thyroglobulin (669 kDa) were loaded onto an 11-ml 5 to 20% sucrose gradient made in a solution containing 0.3 M KCl, 20 mM HEPES (pH 7.9), 2 mM EDTA, 10% glycerol, and 10 mM β-mercaptoethanol. The gradient was centrifuged for 19 h at 40,000 rpm in a Beckman SW41 rotor at 4°C. Fractions (0.5 ml) were taken from the top of the gradient and precipitated with trichloroacetic acid. Samples were either run on a 10% SDS-polyacrylamide gel and Coomassie blue stained for the indicated standards or run on a 6% gel and Western blotted to detect SMCHD1.

### Electron microscopy of negatively stained SMCHD1.

A total of 3.0 μl of a 100-ng/ml solution of purified protein was applied to a freshly glow-discharged carbon-coated copper grid and negatively stained with 0.75% (wt/vol) uranyl formate. Samples were visualized at 80 kV with an FEI T12 electron microscope. Low-dose images were acquired at a ∼0.8-μm underfocus with 15 e^−^/Å^2^ on a high-sensitivity FEI Eagle 4,096- by 4,096-pixel charge-coupled-device (CCD) camera at a nominal magnification of ×46,000, which corresponded to a sampling of 0.27 nm/pixel.

### Cell culture.

Fibroblasts were grown in EC10 medium (Dulbecco's modified Eagle medium [DMEM]; Life Technologies) supplemented with 10% fetal calf serum (FCS; Seralab), 2 mM l-glutamine, 1× nonessential amino acids, 50 μM 2-mercaptoethanol, and 50 μg/ml penicillin-streptomycin (Life Technologies) in a 37°C incubator under 5% CO_2_. ESCs were grown in EC10 medium supplemented with leukemia inhibitory factor (LIF)-conditioned medium at a concentration equivalent to 1,000 U/ml. Stable clonal cell lines were established and maintained under selection with 0.5 μg/ml G418 (Life Technologies).

The following cell lines were used in this study: human HEK293T cells; mouse mammary C127 cells; *Smchd1*^*+/+*^ and *Smchd1*^−/−^ mouse embryonic fibroblasts (MEFs); and the ESC lines E14TG2a, J1, PGK12.1, and *Smchd1*^−/−^ ES23. The *Smchd1*^−/−^ ES23 XY cell line was derived from the inner cell mass (ICM) of an embryonic day 3.5 (E3.5) embryo obtained from an intercross of MommeD1 heterozygote mice (the Smchd1-MommeD1 mutant allele is described in reference 3). WT and *Smchd1*^−/−^ matched MEF cell lines were derived from WT or *Smchd1*-null E9.5 embryos. Transgenic *Smchd1* cell lines were generated by transfection with Lipofectamine (Life Technologies) and selection with G418 antibiotic. Surviving clones were picked, and the SMCHD1-FLAG or SMCHD1-tandem affinity purification (TAP) expression level was analyzed by Western blotting.

### Immunofluorescence.

Cells were split onto slides 16 h before staining at a low density. Slides were then washed in PBS, fixed with 2% formaldehyde in PBS for 15 min, and permeabilized with 0.4% Triton X-100 in PBS for 10 min. After being washed with PBS, the slides were blocked for 1 h in 0.2% fish gelatin (Sigma) in PBS and incubated for 1 h with primary antibody (diluted in 0.2% fish gelatin and 5% normal goat serum). Slides were washed three times in 0.2% fish gelatin and incubated for 1 h with an Alexa Fluor-conjugated secondary antibody (Life Technologies). After two washes in fish gelatin and two washes in PBS, slides were mounted by using mounting medium containing 4′,6-diamidino-2-phenylindole (DAPI) (Vector Laboratories).

### Cell fractionation.

Cell fractionation experiments were performed by using a commercially available subcellular protein fractionation kit (Thermo). NEs for Western blot analyses, peptide pulldown experiments, and immunoprecipitation (IP) assays were prepared according to a modified version of the method described previously by Dignam et al. (15). Briefly, cells were lysed in buffer A (10 mM HEPES [pH 7.9], 1.5 mM MgCl_2_, 10 mM KCl, 0.5 mM DTT, and complete protease inhibitors) with a Dounce homogenizer. Recovered nuclei were resuspended in buffer C (5 mM HEPES [pH 7.9], 10 mM KCl, 26% glycerol, 1.5 mM MgCl_2_, 0.2 mM EDTA, and complete protease inhibitors) supplemented with 125 U Benzonase for 60 min on ice, followed by the addition of 300 mM NaCl and incubation on ice for an additional 30 min. The samples were centrifuged at 13,000 rpm for 20 min at 4°C, and the supernatant was taken as the nuclear extract.

### Immunoprecipitation.

One hundred micrograms of NE (300 mM NaCl) was diluted into IP buffer (50 mM Tris [pH 7.5], 0.05% NP-40) to a final concentration of 150 mM NaCl in 400 μl. A total of 3.0 μl of antibody (see below) and 20 μl of protein A magnetic beads (Life Technologies) were added, and the mixture was incubated for 4 h at 4°C with rotation and then washed four times in IP buffer. Beads were resuspended in 2× Laemmli sample buffer and boiled.

### FLAG immunoprecipitation and mass spectrometry.

Nuclear extracts for mass spectrometry (MS) experiments were prepared as described previously (16, 17). Immunoprecipitation of SMCHD1-FLAG from ESC nuclear extracts using the mouse M2 FLAG antibody (Sigma) and mass spectrometry analysis were carried out as previously described (16). SMCHD1-TAP tag IP from HEK293T cells was performed as described previously (17), and mass spectrometry analysis was performed at the Taplin Biological Mass Spectrometry Facility (Harvard Medical School). Raw data are available for download from our laboratory website or by request (https://sites.google.com/site/brockdorfflab).

### Peptide pulldown assay.

Biotinylated histone peptides containing the N-terminal 41 amino acids of the histone H3 tail, either unmodified or trimethylated on lysines 4, 9, and 27, were purchased (GL Biochem). Twenty micrograms of peptide was mixed with magnetic streptavidin beads (Thermo) in binding buffer (150 mM NaCl, 50 mM Tris [pH 7.5], 0.1% NP-40) for 2 h at 4°C and washed three times in binding buffer. Peptide-conjugated beads were incubated with 400 μg of NE in 400 μl of binding buffer for 4 h at 4°C with rotation and washed four times in binding buffer. Beads were resuspended in 2× Laemmli sample buffer and boiled.

### Western blotting.

Samples were analyzed by 6% or 15% SDS-PAGE and transferred onto polyvinylidene difluoride (PVDF) membranes. Membranes were blocked in 5% milk in Tris-buffered saline (TBS) plus 0.1% Tween 20 (TBST). Membranes were incubated with antibodies in 2.5% milk in TBST and washed in TBST.

### Antibodies.

**FIG 1:**
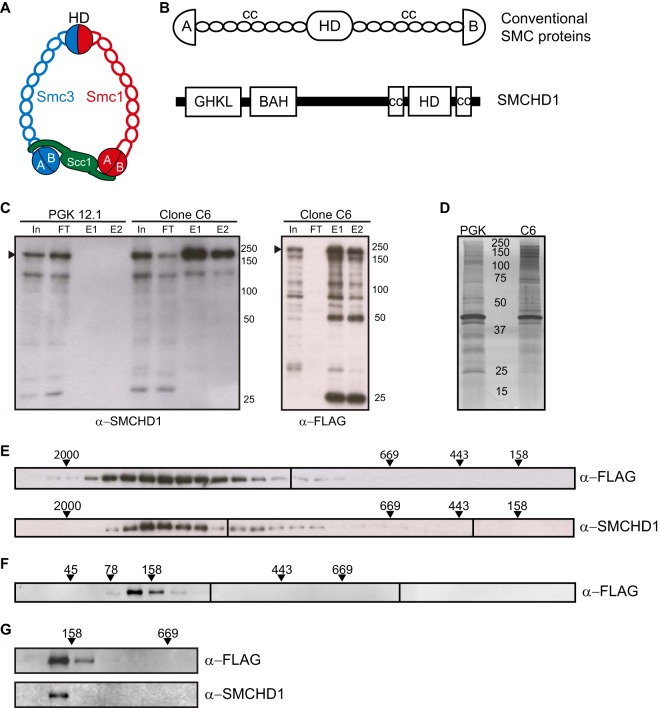
Biochemical characterization of SMCHD1 complexes. (A) Canonical SMC complex, represented here by cohesin, composed of an SMC1-SMC3 heterodimer and an Scc1 (kleisin) subunit. The SMC hinge (HD) and Walker A/B ATPase (A/B) are denoted. (B) Schematics illustrating the domain architecture of canonical SMC proteins and SMCHD1. GHKL ATPase (GHKL), coiled-coil (cc), and bromo-adjacent homology (BAH) domains are denoted. (C) Western blots showing expression of SMCHD1-FLAG or SMCHD1 in parental (PGK12.1) and stable (clone C6) cell lines used in mass spectrometry experiments. Arrowheads indicate full-length endogenous or FLAG-tagged SMCHD1 protein (227 kDa). The four lanes shown for each blot are the input (In), flowthrough (FT), and elutions 1 and 2 (E1 and E2, respectively) from anti-FLAG IP. SMCHD1 is present only in eluents from clone C6. (D) Silver staining of IP material for mass spectrometry from control (PGK) and clone C6 nuclear extracts. (E) Western blot of fractions following size exclusion chromatography of nuclear extracts from C6 clone for FLAG-tagged SMCHD1 (top) and from PGK12.1 ESCs for endogenous SMCHD1 (bottom). (F and G) Western blots of fractions generated from sucrose gradient separation of nuclear extracts from the C6 clone (anti-FLAG) and PGK12.1 (anti-SMCHD1), for all fractions (F) and only selected fractions (G). Molecular mass standards (in kilodaltons) for panels E to G are labeled above each blot.

Antibodies to FLAG (catalog number F3165; Sigma), HA (clone 3F10; Roche), mCherry (catalog number ABE3523; Source Bioscience), green fluorescent protein (GFP) (catalog number ab290; Abcam), HP1α (catalog number MAB3584; Millipore), HP1γ (catalog number MAB3450; Millipore), histone H3 (catalog number ab1791; Abcam), H3K9me2 (catalog number 154-050; Diagenode), H3K9me3 (catalog number 05-1242; Millipore), H3K27me3 (catalog number 61017; Active Motif), and tubulin (catalog number 21445; Cell Signaling) were used in this study. Smchd1 rabbit polyclonal antibody was raised against a mixture of SMCHD1 fragments produced in bacteria (positions 1 to 385, 1197 to 1549, and 1615 to 1963), affinity purified, validated by Western blot analysis (see Fig. 5B), and used for experiments depicted in Fig. 5A, 7D, and 8. Smchd1 antibody (catalog number ab31865; Abcam) was used for experiments depicted in Fig. 1C and E.

### Clustered regularly interspaced short palindromic repeat (CRISPR)/Cas9 mutagenesis.

ES23^+^ or C127 cells were seeded at 1 × 10^5^ cells/ml and transfected on the following day with 1.0 μg of PX459 (18) containing genomic RNAs (gRNAs) for *Lrif1* or *HP1*γ. Transfected cells were selected with either 1.0 or 4.0 μg/ml puromycin (Sigma) for 48 h, followed by recovery and picking of surviving clones. Two guide RNAs for one gene (Table 1) were cotransfected, clones were screened by PCR for genomic DNA deletion, and mutations were confirmed by sequencing. Quantitative RT-PCR (qRT-PCR) was performed to demonstrate the loss of the *Lrif1* transcript by using primers listed in Table 1, and the loss of HP1γ was shown by Western blotting. Several clones of each mutant were tested, and the representative clones shown in the figures are listed in the corresponding legends.

### qRT-PCR.

RNA was prepared with the RNeasy minikit (Qiagen), followed by DNase treatment using a Turbo DNA-free kit (Life Technologies). cDNA was synthesized by using 2.0 μg RNA, using Super-Script III reverse transcriptase (Life Technologies). Quantitative PCR assays were performed on a Rotor-Gene Q instrument (Qiagen), using iQ SYBR green custom supermix (Bio-Rad) and the primers listed in Table 1.

## RESULTS

### Biochemical analysis of SMCHD1 complexes.

Eukaryotic condensin, cohesin, and Smc5/6 complexes form a trimeric ring structure comprised of a heterodimer of two SMC family proteins and a third kleisin subunit (19, 20) (Fig. 1A). SMCHD1 has been classified as an SMC family protein based on the presence of an SMC hinge domain flanked by coiled-coil domains (Fig. 1B). However, the domain organization differs from that of conventional SMC proteins, as the hinge domain is located at the C terminus, rather than centrally, and in that SMCHD1 has a putative GHKL ATPase rather than a Walker A/B ATPase. Additionally, a previous bioinformatic analysis identified a region in SMCHD1, adjacent to the putative GHKL ATPase, that shows homology to the BAH domain family (14) (Fig. 1B).

To identify potential SMCHD1-interacting proteins, we carried out a proteomic analysis using an embryonic stem cell (ESC) line that stably expresses SMCHD1-FLAG at a level similar to that of endogenous SMCHD1 (Fig. 1C). SMCHD1-FLAG was immunoprecipitated from nuclear extracts (16, 17) (Fig. 1C and D), and copurifying proteins were then identified by tandem MS (MS/MS). In addition to SMCHD1, we identified two isoforms of LRIF1 and three HP1 proteins (Table 2; also see the data at https://sites.google.com/site/brockdorfflab). HP1γ and LRIF1 were also identified in a second independent experiment (Table 3; also see the data at https://sites.google.com/site/brockdorfflab), and we therefore conclude that these are genuine SMCHD1 interactors. LRIF1 is the mouse homolog of HBiX1, which was found to interact with both SMCHD1 and HP1 proteins in human cells (7). We did not detect any protein with similarity to the kleisin subunit of canonical SMC complexes, indicating that SMCHD1 probably does not participate in a tripartite ring complex.

**TABLE 2:** SMCHD1 interactors identified by mass spectrometry^*a*^

Protein	Description	Hit	Score	emPAI	Coverage (%)	No. of peptides
SMCHD1	SMC hinge domain-containing 1	1	9,529	17.57	61.4	126
HP1γ	Chromobox homolog 3	20	733	6.39	38.7	9
LRIF1	Ligand-dependent nuclear receptor-interacting factor 1 isoform 1	30	537	0.44	10.6	8
LRIF1	Ligand-dependent nuclear receptor-interacting factor 1 isoform 3	32	525	1.78	31.1	8
HP1α	Chromobox homolog 1	46	342	1.52	23.8	3
HP1β	Chromobox homolog 5	78	190	0.56	12.6	2

a Shown are the names, descriptions, overall ranks (Hit), mascot scores, exponentially modified protein abundance indices (emPAI), percent coverages, and numbers of unique peptides detected for candidate SMCHD1 interactors identified by mass spectrometry (clone G6).

**TABLE 3:** Confirmed SMCHD1-interacting proteins^*a*^

Protein	Description	Hit	Score	emPAI	Coverage (%)	No. of peptides
SMCHD1	SMC hinge domain-containing 1	1	3,067	1.02	27.1	45
LRIF1	Ligand-dependent nuclear receptor-interacting factor 1	78	118	0.12	2.3	1
HP1γ	Chromobox homolog 3	92	85	0.18	9.2	1

a A second mass spectrometry experiment using *Smchd1^−/−^* ESCs stably expressing SMCHD1-FLAG (clone G2) confirms that LRIF1 and HP1γ interact with SMCHD1.

We next analyzed SMCHD1 complexes by fractionation of high-salt nuclear extracts using size exclusion chromatography (SEC) and sucrose gradient analysis. Both endogenous and FLAG-tagged SMCHD1 proteins fractionate in the megadalton mass range by SEC (Fig. 1E), suggesting the existence of a large multimeric complex. However, sucrose gradient analysis demonstrated both endogenous and FLAG-tagged SMCHD1 sediment at ∼100 kDa (Fig. 1F and G), indicating that the native complex is much smaller, with a mass similar to that of an SMCHD1 monomer. A possible explanation for these disparate observations is that SMCHD1 behaves anomalously in SEC experiments, for example, because it adopts a rod-like shape (see also below).

### SMCHD1 forms a stable homodimeric complex.

**FIG 2:**
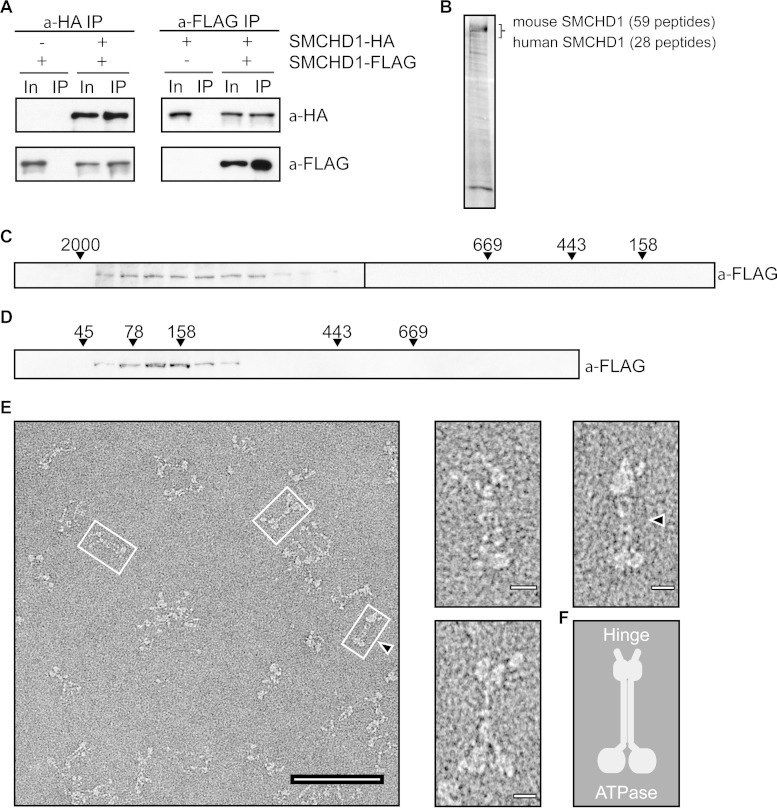
SMCHD1 forms a functional homodimer. (A) Reciprocal immunoprecipitation and Western blotting of SMCHD1-HA and SMCHD1-FLAG from nuclear extracts of HEK293T cells cotransfected with SMCHD1 plasmids. In, input (10%); IP, immunoprecipitate (15%). (B) Silver-stained IP sample from TAP-tagged mouse SMCHD1 expressed in human HEK293T cells. Both human and mouse SMCHD1 peptides were detected at similar levels. (C) Anti-FLAG Western blotting of fractions following size exclusion chromatography of rSMCHD1. (D) Anti-FLAG Western blotting of fractions from sucrose gradient sedimentation of rSMCHD1. Molecular mass standards (kilodaltons) for panels C and D are labeled above each blot. (E) Electron microscopy images of negatively stained rSMCHD1. Selected images are further magnified and presented at the right. Bars, 100 nm (left image) and 10 nm (right images). (F) Schematic of the proposed homodimeric form.

Conventional eukaryotic SMC proteins heterodimerize (21), whereas prokaryotic SMC proteins form homodimers (22). Given that other SMC proteins were not identified in our proteomic analysis (Table 2), we hypothesized that SMCHD1 most likely forms homodimers. To test this, we cotransfected HEK293T cells with SMCHD1 with a C-terminal HA or FLAG tag and then determined their association by immunoprecipitation (IP). As shown in Fig. 2A, IP with anti-HA coprecipitated FLAG-tagged SMCHD1 and vice versa, clearly demonstrating self-association, as either dimers or possibly oligomers. In support of this conclusion, MS/MS analysis of TAP-tagged mouse SMCHD1 expressed in human HEK293T cells identified human-specific SMCHD1 peptides at similar levels (Fig. 2B; see also the data at http://sites.google.com/site/brockdorfflab).

To gain insight into the molecular architecture of SMCHD1, we carried out an analysis of recombinant SMCHD1 (FLAG-rSMCHD1) expressed and purified from insect cells. SEC and sucrose gradient analyses revealed that the properties of FLAG-rSMCHD1 are indistinguishable from those of native SMCHD1 present in nuclear extracts (compare Fig. 1E and F and 2C and D), suggesting that SMCHD1 homodimers represent the predominant functional form *in vivo*. We went on to analyze FLAG-rSMCHD1 by negative-stain electron microscopy (EM) (Fig. 2E). Individual molecules can be observed to possess a rod-like appearance, ∼40 nm in length, with globular domains on either end potentially corresponding to the hinge and ATPase domains. A large proportion of particles were seen to have two linked arms, some closely associated along their entire length (Fig. 2E, arrowheads), indicating homodimerization of individual SMCHD1 subunits. These structures bear a close resemblance in both size and appearance to those in EM micrographs of other SMC complexes, notably homodimeric bacterial condensins (23, 24). Conformational flexibility precluded further refinement of SMCHD1 structures, but the results nevertheless define a probable molecular architecture, illustrated in Fig. 2F.

### The SMC hinge domain mediates SMCHD1 homodimerization.

**FIG 3:**
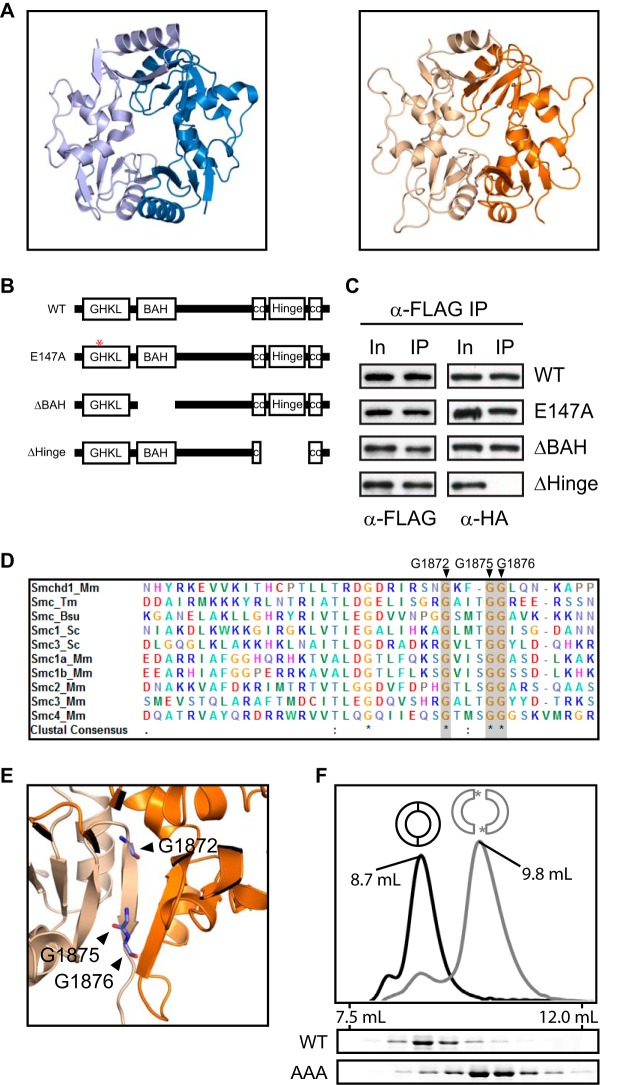
SMCHD1 homodimerizes through the SMC hinge domain. (A, left) Thermotoga maritima Smc hinge dimer structure (PDB accession number 1GXL), shown as light and dark blue monomers. (Right) The SWISS-MODEL-predicted SMCHD1 hinge structure based on the T. maritima hinge is also composed of two monomers, shown in gold and orange. (B) Graphical representation of the FLAG- and HA-tagged SMCHD1 derivatives used in cellular assays. (C) Anti-FLAG immunoprecipitation and Western blotting of SMCHD1 derivatives tagged with either FLAG or HA and cotransfected into HEK293T cells. In, input (10%); IP, immunoprecipitate (15%). (D) ClustalW2 alignment of the hinge domains from several canonical SMC proteins and SMCHD1. Five species are represented in this alignment: Thermotoga maritima (Tm), Bacillus subtilis (Bs), Saccharomyces cerevisiae (Sc), and Mus musculus (Mm). The conserved glycine residues mutated in the SMCHD1 hinge AAA mutant are labeled and highlighted in gray. (E) Hinge dimer ribbon diagram with conserved glycine residues mutated in the SMCHD1 hinge AAA mutant highlighted. These three glycine residues are predicted to be positioned at the interface of the two hinge domain monomers. (F) HD dimerization analysis by size exclusion chromatography. The *A*_280_ peak for the WT (black) and AAA mutant (gray) hinges are shown (top), and the corresponding 0.5-ml fractions were run on an SDS-PAGE gel and Coomassie stained (bottom).

**FIG 4:**
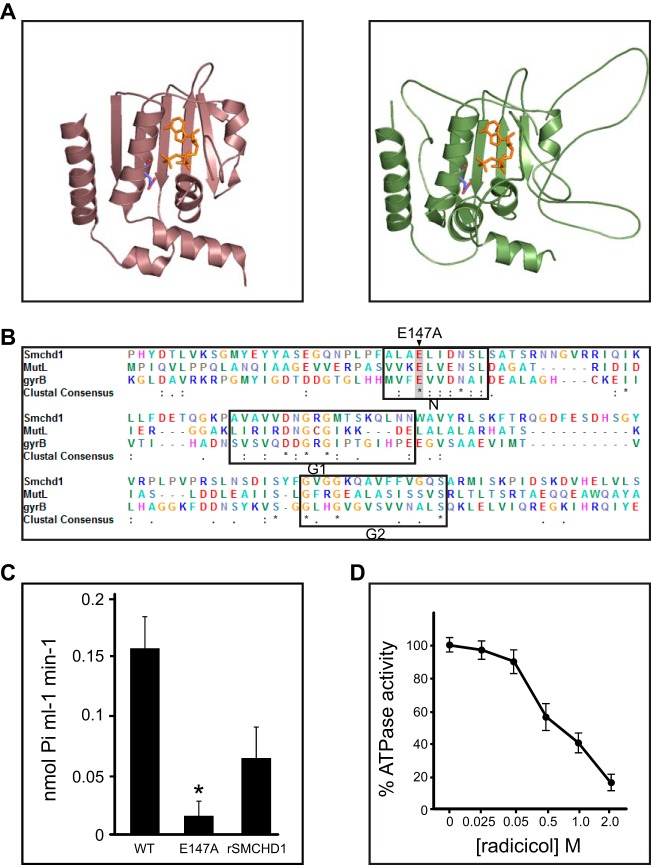
The GHKL ATPase domain hydrolyzes ATP. (A) Structures of MutL homolog 1 (MLH1) from Homo sapiens (PDB accession number 3NA3) (bronze) (left) and the predicted SMCHD1 ATPase domain (green) (right), shown with ATP (orange) and the catalytic glutamic acid (blue/red) highlighted. (B) ClustalW2 alignment of the conserved GHKL ATPase domains from the DNA mismatch repair protein MutL and DNA gyrase subunit B (GyrB) alongside SMCHD1. The conserved N, G1, and G2 motifs are outlined, and the catalytic glutamic acid that is mutated in the SMCHD1 E147A mutant constructs is highlighted in gray. (C and D) ATPase activity assays using purified WT or E147A mutant SMCHD1 ATPase domains and rSMCHD1 (C) or the WT SMCHD1 ATPase domain in the presence of various concentrations of the GHKL ATPase inhibitor radicicol (D). Statistical significance was determined by one-tailed Student's *t* test; the asterisk indicates a *P* value of 0.0017. Results show the mean calculated activities (*n* = 3), and the error bars show standard deviations.

The SMCHD1 HD is homologous to HDs found in both prokaryotic and eukaryotic SMC proteins. Interestingly, phylogenetic analysis indicates that the SMCHD1 HD is more closely related to that found in the prokaryotic SMC proteins, which, like SMCHD1, form homo- rather than heterodimers. A SWISS-MODEL (25, 26) structure homology search identified the HD of the Thermotoga maritima SMC protein (Fig. 3A, left) as being highly similar to the SMCHD1 HD (root mean square deviation [RMSD], 0.102) (Fig. 3A, right). To test the importance of the SMCHD1 HD for dimerization, we cotransfected HEK293T cells with SMCHD1-FLAG and SMCHD1-HA as described above, using a deletion of the entire HD (ΔHinge residues 1642 to 1918) (Fig. 3B). As shown in Fig. 3C, a deletion of the HD results in a loss of SMCHD1 dimerization. In contrast, a deletion of the putative BAH domain (ΔBAH) (Fig. 3B and C) and a mutation abrogating GHKL ATPase activity (Fig. 3B and C and 4) had no effect on SMCHD1 dimerization. In the latter case, it should be noted that the mutant protein can still bind ATP, and in other GHKL family members, this is sufficient for dimer formation (27, 28).

We further analyzed the dimerization of the HD using a recombinant wild-type HD expressed in bacteria (residues 1616 to 1963) or a mutant form with alanine substitutions of three conserved glycines, G1872A, G1875A, and G1876A (referred to as the AAA mutant), which, in prokaryotic HDs, are required for dimer formation (29) (Fig. 3D and E). Dimer formation was analyzed by SEC, as performed previously for other SMC hinge domains (30). As shown in Fig. 3F, wild-type HD forms dimers, but this is abrogated by the use of the AAA mutant, visualized by the slower migration of the monomeric hinge. Together, these findings demonstrate that the SMCHD1 HD mediates homodimerization and that the mechanism is similar to that reported for the HD in canonical SMC proteins.

### The GHKL ATPase domain hydrolyzes ATP.

The GHKL ATPase domain in SMCHD1 is highly homologous to those found in several other proteins, notably DNA gyrase B, HSP90, and MUTL (Fig. 4A and B). Several GHKL domains function as dimers, forming ATP-activated clamps or gates that allow entrapment of the substrate, for example, DNA in the case of DNA gyrase B, MUTL, and topoisomerase II (31).

To test the activity of the putative GHKL domain in SMCHD1, we expressed the conserved region, spanning residues 1 to 385, as a recombinant protein in bacteria and then assayed ATP hydrolysis using a colorimetric assay (Pi ColorLock Gold; Innova Biosciences). As a control, we mutated a conserved glutamic acid residue that is required for catalysis in other GHKL ATPase domains (E147A) (27, 28) (Fig. 4B). As shown in Fig. 4C, WT but not E147A mutant SMCHD1-GHKL hydrolyzes ATP. We confirmed this result using full-length recombinant SMCHD1 (Fig. 4C). Consistent with these observations, the addition of radicicol, a specific inhibitor of the GHKL ATPase family (32, 33), reduced the ATPase activity of the SMCHD1 GHKL domain in a dose-dependent manner (Fig. 4D). Given the molecular architecture of the homodimeric complex determined by EM (Fig. 2E and F), we suggest that, analogous to other GHKL ATPase proteins, SMCHD1 functions as an ATP-activated gate or clamp.

### Role of SMCHD1 conserved domains in chromatin binding.

**FIG 5:**
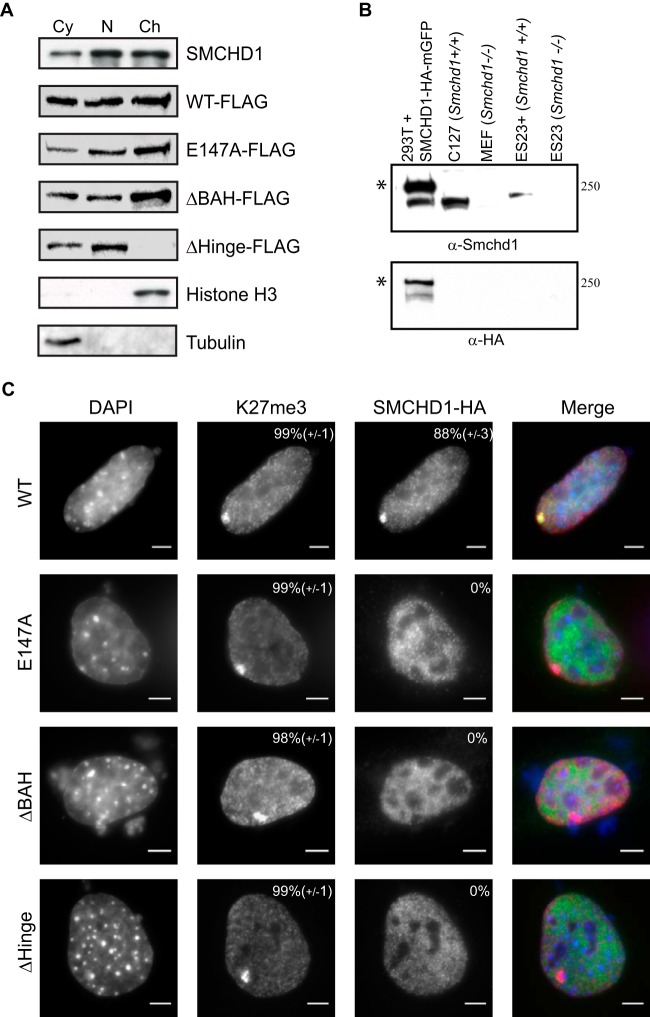
SMCHD1 conserved domains are required for chromatin loading. (A, top) Western blot showing subcellular fractionation of endogenous SMCHD1 and *Smchd1*^−/−^ ES23 cells stably expressing WT SMCHD1-FLAG and mutant derivatives (Fig. 3B) in cytoplasmic (Cy), soluble nuclear (N), and chromatin-bound (Ch) fractions. Endogenous SMCHD1 from WT E14 ESCs is present in all fractions but predominantly in and approximately equally divided between the nuclear and chromatin-bound lanes. (Bottom) Histone H3 as a control for the chromatin-bound fraction and tubulin as a control for the cytoplasmic fraction. (B) Rabbit polyclonal anti-SMCHD1 antibody was tested by Western blotting against nuclear extracts from human HEK293T cells transfected with an SMCHD1-HA-monomeric GFP (mGFP) plasmid, wild-type mouse C127 fibroblasts, *Smchd1*^−/−^ MEFs, *Smchd1*^−/−^ ESCs stably expressing SMCHD1-FLAG (ES23^+^), and *Smchd1*^−/−^ ESCs (ES23). Bands at the predicted molecular masses are visible in extracts containing SMCHD1 but not in extracts from *Smchd1*^−/−^ cells. Asterisks indicate HA-mGFP-tagged protein. (C) Localization of SMCHD1 mutants in *Smchd1*^−/−^ mouse embryonic fibroblasts. Cells were transfected with plasmids containing WT and derivative SMCHD1 constructs and stained with DAPI to mark DNA (blue), anti-K27me3 antibody for Xi (red), and anti-HA for SMCHD1 (green). The percentage of cells showing a focus for Xi is shown at the top right of each antibody panel, representing the means from 3 replicates (*n* > 50 cells). Bars, 5 μm.

To investigate the role of conserved domains in SMCHD1, we first analyzed the distribution of SMCHD1 in soluble compared to chromatin-bound nuclear extracts by using *Smchd1*-null ESCs stably expressing either WT SMCHD1-FLAG (here referred to as ES23^+^) or domain mutant proteins as described above. As shown in Fig. 5A, WT SMCHD1-FLAG shows similar distributions between chromatin-bound and soluble fractions, as does endogenous SMCHD1 (Fig. 5A and B). Deletion of the BAH domain or mutation of the GHKL ATPase had no effect on chromatin association. Deletion of the HD, on the other hand, resulted in the complete dissociation of SMCHD1 from the chromatin-bound fraction. The latter observation may indicate that the HD mediates direct or indirect chromatin binding or, alternatively, that SMCHD1 dimerization is important for this interaction (see also below).

SMCHD1 plays an important role in gene silencing in X chromosome inactivation, and consistent with this, the SMCHD1 protein is strongly enriched over the Xi territory in differentiated XX somatic cells (3, 6, 7). To determine the importance of SMCHD1 conserved domains for Xi localization, we transfected WT and mutant SMCHD1-HA expression constructs into *Smchd1*^−/−^ XX mouse embryonic fibroblasts (MEFs). As illustrated in Fig. 5C, WT SMCHD1-HA localizes to Xi territories, as defined by costaining with antibody against the Xi-enriched histone modification H3K27me3 (34, 35). However, a mutation affecting the activity of the GHKL ATPase and deletion of the BAH domain result in the complete loss of Xi localization (Fig. 5C). This contrasts with the effect of these mutations on the chromatin association of SMCHD1, as determined by nuclear fractionation experiments (Fig. 5A). SMCHD1 lacking the HD also failed to localize to Xi. The latter observation was expected given the complete dissociation of the HD mutant protein from chromatin (Fig. 5A).

### Interaction of SMCHD1 with histone H3K9me3.

**FIG 6:**
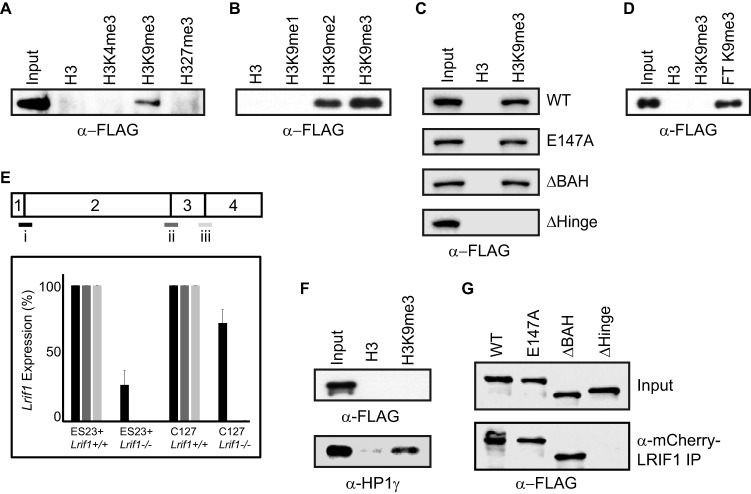
LRIF1 mediates indirect interaction of SMCHD1 with H3K9me3-modified chromatin. (A to D) Western blots of bound proteins following peptide pulldown experiments on nuclear extracts from ES23^+^ ESCs with unmodified H3, H3K4me3, H3K9me1/2/3, and H3K27me3 peptides (A and B) and on nuclear extracts from ES23 ESCs complemented with WT or mutant SMCHD1-FLAG derivatives and H3K9me3 peptide (C) and full-length recombinant SMCHD1 (rSMCHD1) and H3K9me3 peptide (D). The flowthrough (FT) lane shows that rSMCHD1 remains in solution and is not bound to the H3K9me3 peptide. (E) Quantitative RT-PCR to verify the loss of transcript in *Lrif1*^−/−^ cell lines created by CRISPR mutagenesis of exon 3/4. A schematic of the *Lrif1* coding sequence is drawn with numbered exons, with three primer sets spanning intron/exon boundaries for qRT-PCR below (i to iii). *Lrif1* expression in the mutant clones is shown as a percentage relative to the expression level in the wild-type parental cell line. Both the ES23^+^ and C127 *Lrif1*^−/−^ cell lines show reduced transcript levels with primer set i and a loss of transcript with primer sets ii and iii. (F) Western blots for SMCHD1-FLAG and HP1γ (control) following peptide pulldown experiments with nuclear extracts from *Lrif1*^−/−^ ES23^+^ ESCs. (G) Anti-FLAG Western blotting with mCherry-LRIF1 immunoprecipitation of nuclear extracts from HEK293T cells cotransfected with mCherry-LRIF1 and SMCHD1-FLAG derivatives. Input, 10%; IP, 30%.

Independent proteomic screens identified SMCHD1 as interacting with the histone modification H3K9me3 (36, 37). To further investigate the molecular basis of this observation, we performed peptide pulldown on nuclear extracts from ES23^+^ cells and assessed the binding of SMCHD1 with histone H3 tail peptides methylated at different lysine sites. As shown in Fig. 6A, SMCHD1 bound to H3K9me3 but not to unmodified H3, H3K4me3, or H3K27me3. Additionally, we observed that SMCHD1 interacts with H3K9me2 albeit to a lesser extent and does not interact with H3K9me1 (Fig. 6B).

We next determined if mutation of SMCHD1 conserved domains affects H3K9me3 binding by performing peptide pulldown experiments on nuclear extracts from *Smchd1*^−/−^ ESCs complemented with either WT or mutant *Smchd1*. In particular, we were interested to test the role of the BAH domain, which in other proteins has been shown to bind nucleosomes (38), histone H3 (39), or specific histone lysine methylation sites, including H3K9me3 (40, 41). However, as shown in Fig. 6C, we found that neither the BAH domain nor SMCHD1 GHKL ATPase activity is required for H3K9me3 binding. Deletion of the HD, on the other hand, resulted in the loss of binding to H3K9me3, indicating that either this region mediates H3K9me3 binding or SMCHD1 dimerization is important for the interaction.

We failed to detect binding to H3K9me3 using full-length FLAG-rSMCHD1 in the peptide pulldown assay (Fig. 6D), suggesting that the interaction is likely to be indirect. A previous study demonstrated that HBiX1, the human homolog of LRIF1, interacts with the HP1 family of proteins that bind to H3K9me3 as well as with human SMCHD1. Moreover, we identified both LRIF1 and HP1γ proteins as being SMCHD1 interactors (Tables 2 and 3). To determine if LRIF1 mediates SMCHD1 binding to H3K9me3, we generated *Lrif1*-null ES23^+^ ESCs using CRISPR/Cas9 genome editing (see Materials and Methods) (Fig. 6E) and then, using nuclear extracts from these cells, determined the interaction with H3K9me3 peptides. As shown in Fig. 6F, mutation of *Lrif1* entirely abolishes H3K9me3 binding of SMCHD1 but does not abolish binding of HP1γ. Additionally, by analysis of HEK293T cells cotransfected with mCherry-LRIF1 and SMCHD1/SMCHD1 mutants, we determined that only the HD region is required for the interaction of SMCHD1 and LRIF1 (Fig. 6G). This observation is consistent with the finding that HD deletion abrogates H3K9me3 binding (Fig. 6C).

### Autonomous pathways mediate SMCHD1 loading at H3K9me3 sites and on Xi.

**FIG 7:**
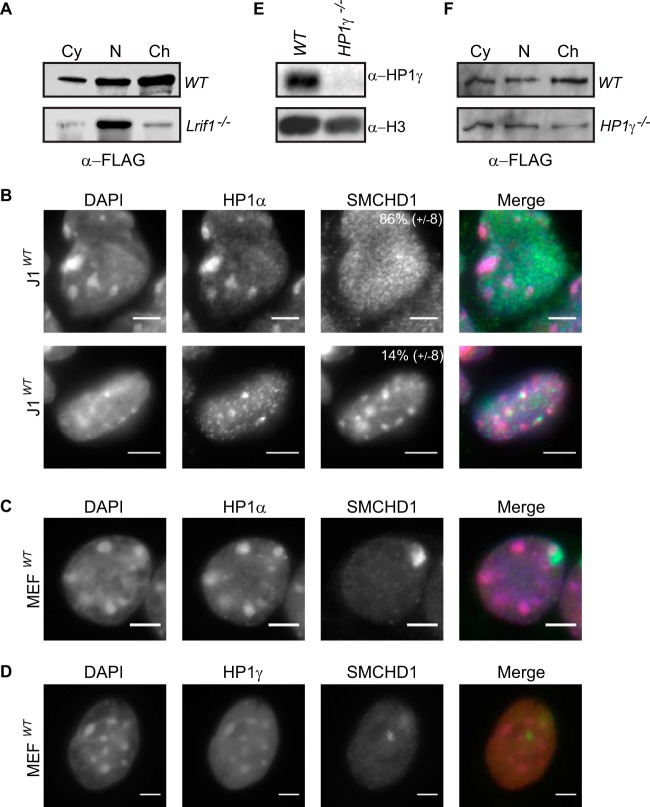
SMCHD1 does not predominantly colocalize with HP1 proteins at pericentric regions. (A) Western blot showing the SMCHD1-FLAG distribution following subcellular fractionation of WT and *Lrif1*-null (clone G5) ES23^+^ cells into cytoplasmic (Cy), soluble nuclear (N), and chromatin-bound (Ch) fractions. (B) SMCHD1 staining in WT J1 XY ESCs. Pericentric heterochromatin domains are visualized with HP1α and are also visible in DNA (DAPI) panels. The percentage of cells that contain either broad nuclear staining (top) or pericentric staining (bottom) are listed at the top right corner of the SMCHD1 panels. Scoring data are the means from 3 replicates (*n* > 150 cells). (C and D) SMCHD1 staining in WT MEFs. (C) Staining for HP1α is enriched at pericentric heterochromatin domains but not Xi, which is enriched for SMCHD1. (D) HP1γ enrichment at pericentric foci and also throughout the chromatin arms is seen in a broad staining pattern more similar to that of SMCHD1. Note the two Xi foci in panel D. Bars, 5 μm. (E) Western blotting for HP1γ on nuclear extracts from WT and *HP1*γ-null (clone H5) ES23^+^ cells. Histone H3 is shown as a control. (F) Western blot showing the SMCHD1-FLAG distribution following subcellular fractionation of WT and *HP1*γ-null (clone H5) ES23^+^ cells.

To determine the importance of the LRIF1-mediated interaction of SMCHD1 and H3K9me3, we analyzed the effect of *Lrif1* mutation on the distribution of SMCHD1 in different nuclear fractions in ES23^+^ cells. As shown in Fig. 7A, loss of *Lrif1* results in a dramatic redistribution of most, although not all, of the SMCHD1 from the chromatin-bound to soluble nuclear fractions. This result suggests that LRIF1 functions as a loading factor directing SMCHD1 to H3K9me3-modified chromatin, likely through the interaction of LRIF1 with both the SMCHD1 and HP1 proteins. However, analysis of SMCHD1 localization by immunofluorescence indicates a more complex picture in relation to H3K9me3. Specifically, we observed SMCHD1 at pericentric heterochromatin domains, major sites of H3K9me3 accumulation, in only a minority of cells (Fig. 7B). The majority of cells exhibit broad pannuclear SMCHD1 staining and, in female somatic cells, a single focus corresponding to Xi (Fig. 7B and C).

A possible explanation is that LRIF1 mediates the interaction of SMCHD1 specifically with HP1γ, which, in contrast to HP1α/β, localizes strongly to H3K9me3 sites on chromosome arms (42, 43) (Fig. 7D). Consistent with this suggestion, HP1γ was the predominant isoform identified in proteomic analyses of SMCHD1-interacting proteins (Tables 2 and 3). To test this hypothesis, we generated HP1γ-null ES23^+^ ESCs by CRISPR/Cas9 mutagenesis (Fig. 7E) and then analyzed the SMCHD1-FLAG distribution by a cell fractionation assay. Our results show a shift of SMCHD1-FLAG from the chromatin fraction to the soluble nuclear fraction (Fig. 7F), although the effect is not as strong as that in *Lrif1*-null cells (Fig. 7A). Together, our observations suggest that LRIF1 mediates the loading of SMCHD1 at sites where HP1 is bound to H3K9me3, notably on the chromosome arms.

**FIG 8:**
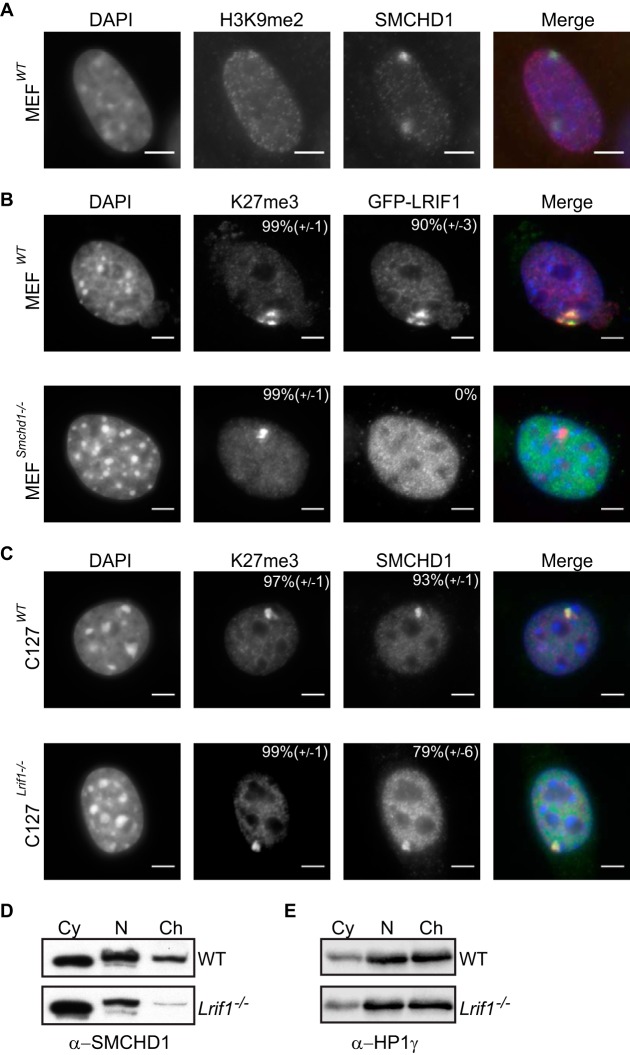
SMCHD1 chromatin loading onto Xi is independent of LRIF1. (A) H3K9me2 and SMCHD1 accumulation on the inactive X chromosome in wild-type mouse MEFs. Note the two foci. (B) Localization of GFP-LRIF1 in WT and *Smchd1*-null MEFs. Cells are stained for DNA (DAPI) (blue), Xi (H3K27me3) (red), and GFP-LRIF1 (anti-GFP) (green). (C) Localization of SMCHD1 in WT and *Lrif1*-null (clone G6) C127 cells. Cells are stained for DNA (DAPI) (blue), Xi (H3K27me3) (red), and SMCHD1 (anti-SMCHD1) (green). The percentage of cells showing a focus for Xi is shown at the top right of each antibody panel, representing the means from 3 replicates (*n* > 50 cells [B] and *n* > 200 cells [C]). Bars, 5 μm. (D and E) Cell fractionation and Western blotting for endogenous SMCHD1 in WT and *Lrif1*-null (clone G6) C127 cells. HP1γ is shown as a control.

In human cells, Xi shows enrichment of H3K9me3 within specific subdomains, and SMCHD1 localization has been linked to the recruitment of HBiX1/SMCHD1 (7). However, there is no detectable enrichment of H3K9me3 on Xi in mouse cells, and although there is an accumulation of H3K9me2 (44) (Fig. 8A), the developmental kinetics are quite distinct from those observed for SMCHD1 (3, 6). To further investigate the relationship between LRIF1 and SMCHD1, we transfected GFP-tagged LRIF1 in WT and *Smchd1*^−/−^ XX MEFs. As shown in Fig. 8B, LRIF1 localizes to Xi in WT cells but not in *Smchd1*^−/−^ cells. This result suggests that the enrichment of LRIF1 on Xi is attributable to the interaction with SMCHD1 rather than binding of H3K9me2 or other modifications present on Xi.

It is possible that LRIF1 and SMCHD1 have an interdependent relationship with regard to Xi localization in mouse cells, as previously suggested for human cells (7). To test this possibility, we generated *Lrif1*^−/−^ C127 XX somatic cells using the same CRISPR/Cas9 strategy as the one described above (Fig. 6E). As shown in Fig. 8C, SMCHD1 localizes to the inactive X territory in the absence of LRIF1. Nucleoplasmic staining for SMCHD1 was increased in *Lrif1*^−/−^ cells relative to WT controls, presumably a by-product of the dissociation from genome-wide chromatin targets. Consistent with this hypothesis, we observed that the majority of SMCHD1 dissociates from chromatin in *Lrif1*^−/−^ C127 cells (Fig. 8D), similar to our observation of ESCs (Fig. 7A). The HP1γ association with chromatin, on the other hand, was unaffected in *Lrif1*^−/−^ cells (Fig. 8E). Taken together, these results demonstrate that two distinct pathways determine SMCHD1 loading at chromatin target sites. GHKL ATPase/BAH-independent binding to H3K9me3 modified nucleosomes via interaction of SMCHD1 with LRIF1/HP1 and the GHKL ATPase/BAH-dependent association with Xi.

## DISCUSSION

### Biochemical properties of SMCHD1.

Through a direct analysis of proteins that interact with SMCHD1, we identified LRIF1 and HP1γ, an observation that is consistent with a previous study that identified human SMCHD1 as an interactor of the LRIF1 homolog HBiX1 (7). However, we find no evidence for major interacting proteins. Moreover, native SMCHD1 and recombinant SMCHD1 behave very similarly in gel filtration and sucrose density gradient analyses. In this regard, SMCHD1 is distinct from other SMC complexes, all of which include an essential stoichiometric kleisin subunit essential for function. Interestingly, despite this fundamental difference, EM micrographs of SMCHD1 homodimers bear a striking resemblance to those reported for canonical SMC proteins. We conclude that the SMCHD1 homodimer most likely represents the core functional complex in an *in vivo* context.

We show that the hinge domain of SMCHD1 mediates the homodimerization of the protein, a finding that is consistent with a very recently reported study demonstrating that the SMCHD1 hinge domain forms dimers (45) and with the similarity of the SMCHD1 hinge domain to canonical bacterial SMC proteins, which also form homodimers. We also demonstrate that the SMCHD1 GHKL ATPase hydrolyzes ATP and, based on analogy with other GHKL ATPase proteins, likely functions as a molecular clamp or pincer.

In addition to the hinge and GHKL domains, SMCHD1 has a domain that shares weak homology with the BAH domain, found in several chromatin binding/modifying proteins. The role of the SMCHD1 BAH domain is not clear at present, although our data suggest that it does not mediate interactions with specific histone H3 tail modifications, notably H3K9me3, as has been reported for some other BAH domains (40, 41). Our data also suggest that the BAH domain is not required for general chromatin associations, as the deletion mutants localize with the chromatin fraction in nuclear extracts. We cannot rule out that the BAH domain binds to other untested histone tail modifications or that it is required for targeting to specific chromatin regions. However, we favor an alternative model in which the BAH domain plays a structural role required for the catalytic cycle of the SMCHD1 protein, similar to the previously suggested role for the BAH domain in the DNA methyltransferase DNMT1 (46). Taking into consideration data from our biochemical analysis, we speculate that the SMCHD1 dimer clamps the chromatin fiber and, through GHKL ATPase activity, catalyzes dynamic changes in chromatin organization, for example, by bringing together distant sites, either in *cis* or in *trans*.

### Interaction of SMCHD1 with histone H3K9me3.

We observed a specific interaction of SMCHD1 with H3K9me3, confirming previously reported observations (36, 37), and further show that this interaction is indirect, mediated by LRIF1 binding to both the SMCHD1 and HP1 proteins. Analysis of SMCHD1 mutants indicates that LRIF1/H3K9me3 binding requires the hinge domain but neither the BAH domain nor the GHKL ATPase activity of the complex. It was previously shown that a coiled-coil domain at the C-terminal end of the LRIF1 homolog HBiX1 mediates interactions with SMCHD1 (7). This domain could potentially interact with the SMCHD1 HD, although we think that it is more likely that the interaction occurs between the LRIF1 coiled-coil and the short coiled-coil domains that flank the SMCHD1 HD (Fig. 1B).

Our *in vitro* analysis demonstrates that LRIF1 mediates the interaction of SMCHD1 with H3K9me3, but somewhat paradoxically, SMCHD1 shows a broad nuclear localization with a concentrated signal over Xi and usually not over pericentric heterochromatin domains, the major sites of H3K9me3 deposition and HP1 binding. A possible explanation, suggested by our proteomic analysis, is that SMCHD1/LRIF1 preferentially interacts with HP1γ, which, unlike other HP1 paralogs, localizes extensively to H3K9me3 on chromosome arms in addition to pericentric heterochromatin domains (42, 43). While our cell fractionation experiments in HP1γ-null cells reveal some functional overlap of HP1α/β regarding SMCHD1 bulk chromatin loading, this result remains consistent with the hypothesis that SMCHD1/LRIF1 complexes favor HP1γ. A similar preference for HP1γ was evident in proteomic data obtained for the HBiX1 interactome (7). Moreover, defined loci that are regulated by HP1γ binding to H3K9me3, notably telomeres (47) and the D4Z4 locus in FSHD patients (48), have also been identified as SMCHD1/LRIF1 targets (9, 49). These considerations suggest that SMCHD1 is an important downstream effector of HP1γ at H3K9me3 sites on the chromosome arms.

### Chromatin loading of SMCHD1.

**FIG 9:**
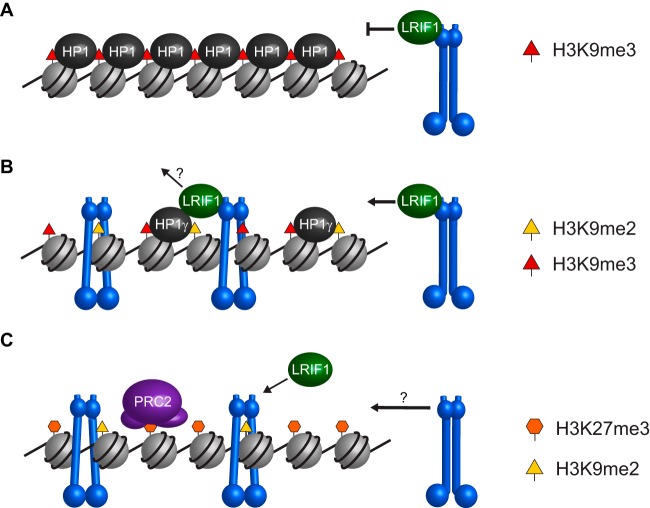
Model for SMCHD1 recruitment to chromatin. (A) Constitutive heterochromatin such as that found at pericentromeric regions is marked by H3K9me3 and all three HP1 paralogs. Recruitment of SMCHD1 (blue) to these sites by LRIF1 may be blocked by the compact and inaccessible organization of HP1 protein oligomers. (B) Other heterochromatic sites, such as telomeres and subtelomeric D4Z4 repeats, are marked by H3K9me2 and H3K9me3 and HP1γ. SMCHD1 recruitment to these sites is mediated by LRIF1 and is independent of the ATPase and BAH domains. (C) SMCHD1 recruitment to Xi in mouse, which is marked with H3K27me3 and H3K9me2, is independent of LRIF1 but requires ATPase activity and the BAH domain. The molecular mechanism behind SMCHD1 loading onto Xi is unknown but may be an active process similar to the loading of conventional SMC proteins via hinge opening.

Nuclear fractionation analysis demonstrates that a major pool of SMCHD1 protein is stably bound to chromatin. Neither GHKL ATPase activity nor the BAH domain is required for this association. However, deletion of LRIF1 or of the SMCHD1 hinge domain region that is required for LRIF1 interactions results in a redistribution of the bulk of SMCHD1 to the soluble nucleoplasm. These findings suggest that LRIF1, in conjunction with HP1γ located principally on chromosome arms, functions as an SMCHD1 loading complex (Fig. 9B). This may be analogous to the role of Scc2/4 in loading the cohesin complex (50). We suggest that LRIF1-dependent loading establishes an initial association of SMCHD1 with chromatin that is subsequently maintained independently of LRIF1. One possibility is that loading results in a topological trapping of the chromatin fiber by the SMCHD1 dimer, similar to cohesin and possibly other canonical SMC complexes (Fig. 9B). We further suggest that the GHKL ATPase and/or the BAH domain plays a role in SMCHD1 dynamics/unloading, as neither is required for the stable association of SMCHD1 with chromatin.

While the LRIF1/HP1-mediated interaction with H3K9me3 appears to be the primary mechanism for chromatin loading of SMCHD1, our findings demonstrate that a completely independent loading pathway accounts for SMCHD1 localization to Xi in mouse cells (Fig. 9C). Specifically, we find that LRIF1 localization to Xi is dependent entirely on SMCHD1 and, conversely, that SMCHD1 localizes to Xi independently of LRIF1. These results indicate that LRIF1-mediated recognition of H3K9me3 does not significantly contribute to SMCHD1 Xi enrichment. The molecular basis for the alternative loading pathway is unclear, although both GHKL ATPase activity and the BAH domain are required, suggesting that SMCHD1 turnover/dynamics may underpin Xi localization. Analysis of the dynamics of SMCHD1 associations with Xi and other sites may be applied to test this idea in the future. Additionally, it will be interesting to determine whether SMCHD1 recruitment to other target sites, for example, the protocadherin and FSHD loci, depends on the LRIF1 or alternative loading pathways.

In summary, our results provide important insights into the molecular mechanisms for loading the chromosomal protein SMCHD1 at different sites in the genome. The LRIF1/H3K9me3 loading pathway highlights the role of SMCHD1 as an important downstream effector at HP1 target sites, with implications for understanding the regulation of heterochromatin at diverse loci and in biological processes that include telomere function, silencing of transposable elements/repeat sequences, and heritable gene silencing.
